# Higher pharmaceutical public expenditure after direct price control: improved access or induced demand? The Colombian case

**DOI:** 10.1186/s12962-018-0092-0

**Published:** 2018-03-02

**Authors:** Sergio I. Prada, Victoria E. Soto, Tatiana S. Andia, Claudia P. Vaca, Álvaro A. Morales, Sergio R. Márquez, Alejandro Gaviria

**Affiliations:** 1 Centro PROESA, Universidad Icesi, Calle 18 No. 122-135, Cali, Colombia; 2 Facultad de Ciencias Sociales, Universidad de los Andes, Carrera 1 # 18A-12, Bogotá, Colombia; 3 Centro de Pensamiento Medicamentos, Información y Poder, Facultad de Ciencias/Departamento de Farmacia, Universidad Nacional, Bogotá, Colombia; 4 Ministerio de Salud y Protección Social, Carrera 13 # 32-76, Bogotá, Colombia

**Keywords:** Pharmaceutical price control, Public expenditure, Developing countries, Middle income

## Abstract

**Background:**

High pharmaceutical expenditure is one of the main concerns for policymakers worldwide. In Colombia, a middle-income country, outpatient prescription represents over 10% of total health expenditure in the mandatory benefits package (POS), and close to 90% in the complementary government fund (No POS). In order to control expenditure, since 2011, the Ministry of Health introduced price caps on inpatient drugs reimbursements by active ingredient. By 2013, more than 400 different products, covering 80% of public pharmaceutical expenditure were controlled. This paper investigates the effects of the Colombian policy efforts to control expenditure by controlling prices.

**Methods:**

Using SISMED data, the official database for prices and quantities sold in the domestic market, we estimate a Laspeyres price index for 90 relevant markets in the period 2011–2015, and, then, we estimate real pharmaceutical expenditure.

**Results:**

Results show that, after direct price controls were enacted, price inflation decreased almost − 43%, but real pharmaceutical expenditure almost doubled due mainly to an increase in units sold. Such disproportionate increase in units sold maybe attributable to better access to drugs due to lower prices, and/or to an increase in marketing efforts by the pharmaceutical industry to maintain profits.

**Conclusions:**

We conclude that pricing interventions should be implemented along with a strong market monitoring to prevent market distortions such as inappropriate and unnecessary drug use.

## Background

Colombia is the first country in Latin-American to achieve nearly Universal Health Coverage, offering a benefit package (POS, henceforth per the Spanish acronym) to all citizens regardless of whether they contribute or not [1]. The package includes services and technologies for health promotion, prevention, diagnosis, treatment and rehabilitation services for all levels of complexity [2]. In addition, all citizens have access to technologies and social services excluded from POS (No-POS, henceforth per the Spanish acronym) through two mechanisms: (i) by requesting that a scientific technical committee studies the pertinence of using a new technology that supposedly it’s better than those included in the benefit package (POS), (ii) by a judicial mandate that orders the State to pay directly for the new technology in protection to the individual’s constitutional right to health.

The former mechanism was abolished in early 2017. Currently prescribers must fill in an electronic form in order to get direct approval by the government. Under any mechanism, new and current technologies and services, not included in the package of benefits (POS), are paid for by the government under a reimbursement scheme. Insurers pay providers and then request reimbursement to a central fund, which audits and decides whether to reimburse totally or partially according to rules defined by the government.

Hence, rising pharmaceutical expenditure is a great concern for health authorities, given both its importance in the total health expenditure and its rapid growth [3]. In 2008, for example, it represented 10.2% of POS expenditure [4] and close to 90% of No-POS expenditure [5]. While POS expenditure has been stable and even decreasing [4], No-POS expenditure is seriously threatening the health system’s financial stability [5, 6]. In 2004, reimbursements paid were a mere US 34 million, in 2007 US 207m, and in 2010 reached US 828m [7, 8]. Since 2010 the amount paid by the government remains around US 840m due to budgetary constraints, but the amount claimed by insurers is about 25% more. The concentration of this pharmaceutical expenditure is also impressive, in 2011, only 38 drugs accounted for 88% of these reimbursements [5].

By legal requirement, POS benefits must be updated every other year, mostly by including new technologies [9]. However, the new inclusions have not halted the No-POS reimbursement requests. A good reason for that is that No-POS expenditure is mainly for new technologies for high-cost diagnosis such as Cancer, Arthritis, HIV, rare diseases and organ transplants. There is also considerable No-POS expenditure on brand-name drugs excluded from POS and on social services such as diapers for older adults [6]. In addition, during the previous decade, Colombia had the highest pharmaceutical prices in Latin America due to the lack of pharmaceutical regulatory framework [10]. Reimbursement prices for No-POS drugs were comparatively higher in Colombia than that in other country in the region [10].

In the context of this growing pressure to control No-POS pharmaceutical expenditure, the government enacted a comprehensive pharmaceutical policy that includes various tools depending on the policy objectives. There are two different institutions involved in pharmaceutical price regulation. The *Comisión Nacional de Precios de Medicamentos y Dispositivos Médicos* (CNPMDM),^1^ an inter-ministerial commission, oversees the design of the methodology whereby drug prices are to be regulated. The other institution is the Ministry of Health (MOH), responsible for implementing price regulation.

Two different mechanisms were used initially by the MoH and the CNPMDM to regulate the pharmaceutical market: price caps on No-POS drugs to be reimbursed by the government, and the introduction of an external price referencing (ERP) system for selected therapeutical groups.

Price caps for No-POS drugs were mainly used between 2010 and 2012. The CNPMDM established a maximum price for No-POS drugs [8] at which the government reimburses the health insurance company. The cap was calculated as the median wholesale price less a fixed percentage (20%). In 2011, around 80 commercial drugs were regulated using this methodology [11].

In 2013, the CNPMDM introduced ERP. If the price of a selected drug was higher in Colombia than that of the 25 percentile within a set of 17 countries where the drug was already commercialized, the price was regulated setting the price to the 25 percentile [8]. A drug was eligible for regulation when: (1) it was considered of public interest for public health reasons or because it had a high financial impact and (2) it had no therapeutic substitutes or it had a high market concentration when compared to other drugs in the same therapeutic group. Both POS and No-POS drugs could be regulated and the regulation applied to market prices, and not only to government reimbursement prices like the previous cap prices [12].

Overall, the CNPMDM regulated 79 ATC using ERP between 2013 and 2014 [13–16]. By 2013, the ERP scheme included more than 3000 products, covering 80% of public drug expenditure [17].

In addition to direct control pricing, the government put into practice another measure in order to control health expenditure and increase access. By including in the benefit package (POS) some of the highest reimbursed No-POS drugs, the government expected that payers would be able to bargain prices down. Between 2011 and 2015 the benefit package was updated twice, in 2011 and 2013.

In this context, the aim of this study is to investigate the effects of the Colombian policy efforts to contain pharmaceutical expenditure by controlling prices. Numerous studies have analyzed the types of ERP and their impacts on outcomes in industrialized countries [18–21], but there are scant analyses of similar issues in middle income countries such as Colombia [22, 23]. Similarly, despite its prominent application, only few studies have analyzed the impact of EPR on outcomes [24].

The paper thus contributes to the existing literature by providing evidence of ERP application in a middle-income country and by estimating real pharmaceutical expenditure effects based on a Laspeyres price index for 90 relevant markets in the period 2011–2015. Also, given that the increase or decrease in pharmaceutical expenditure is a result of the simultaneous variation in price and quantity [3], the paper decomposes pharmaceutical spending growth into price and quantity components, to better understand the direct and indirect effects of price regulation of pharmaceuticals in Colombia. The paper concludes with some general policy recommendations for low and middle-income countries.

## Study data and methods

### Data

Data on prices and quantities sold comes from *Sistema de Información de Precios de Medicamentos* (SISMED). Anyone selling pharmaceuticals (i.e., the pharmaceutical industry, wholesalers, health providers and public facilities) must report prices and quantities sold by type of market. SISMED contains monthly data on purchased price, sales price, sold units, ATC, name drug-brand, pharmaceutical form, minimum units of concentration (UMC), type of market and type of seller (laboratory, wholesaler, insurer and provider). SISMED distinguishes two distinct markets [18]: institutional and private. The institutional market involves public resources and agglutinates public and private entities. The private market is the OTC market. Governmental price controls apply only to the institutional market.

## Methods

For this study, the top 90 drugs by sales in 2015 at the active ingredient level were selected. Criteria for selection were whether the market was regulated, whether there were substitutes, and whether the drug in question had been included in POS updates. Data on prices and quantities were available monthly since 2011 through 2015 (see Table 1). To estimate real pharmaceutical expenditure, we computed a monthly price index for the period 2011–2015. There are several types of price (inflation) indexes. The three most common are Laspeyres, Paasche and Fisher. A Laspeyres price index is calculated as an arithmetic mean of a fixed bundle of goods and services. A Paasche index is a ratio that compares the total cost of a bundle of goods and services valued at current prices with the value of that same bundle at base-period prices. A Fisher index is the geometric mean of a Laspeyres and a Paasche index (that prices a fixed bundle of current period goods and services). Due to reporting missing values on quantities in several months [3], we decided to use a Laspeyres price index:

**Table 1:** Molecules analysed

ATC	ATC name	ATC regulated
A10AB01	INSULINA (HUMANA)	Non regulated
A10AB04	INSULINA LISPRO	Regulated
A10AB06	INSULINA (HUMANA)	Regulated
A10AC01	INSULINA (HUMANA)	No regulated
A10AD01	INSULINA (HUMANA)	No regulated
A10AD04	INSULINA LISPRO	Regulated
A10AE04	INSULINA GLARGINA	Regulated
B01AC04	CLOPIDOGREL	Regulated
B01AC05	TICLOPIDINA	Non regulated
B01AC06	ACETILSALICILICO ACIDO	Regulated
B01AC07	DIPIRIDAMOL	Regulated
B01AC09	EPOPROSTENOL	Non regulated
B01AC11	ILOPROST	Regulated
B01AC13	ABCIXIMAB	Non regulated
B01AC17	TIROFIBAN	Regulated
B01AC22	PRASUGREL	Regulated
B01AC23	CILOSTAZOL	Non regulated
B02BD02	COAGULACION FACTORES VIII	Regulated
B02BD03	FACTOR VIII INHIBIDOR ACTIVADO POR BYPASS	Regulated
B02BD04	COAGULACION FACTOR IX	Regulated
B02BD06	COAGULACION FACTORES VIII	Regulated
B02BD07	COAGULACION FACTOR XIII	Regulated
C07AB02	METOPROLOL	Regulated
C07AB12	NEBIVOLOL	Non regulated
C07AG01	LABETALOL	Regulated
C07AG02	CARVEDILOL	Regulated
C10AA01	SIMVASTATINA	Regulated
C10AA02	LOVASTATINA	Regulated
C10AA03	PRAVASTATINA	Regulated
C10AA04	FLUVASTATINA	Non regulated
C10AA05	ATORVASTATIN	Regulated
C10AA05//C10AX09	ATORVASTATIN//EZETIMIBE	Regulated
C10AA07	ROSUVASTATINA	Regulated
G04CA01	ALFUZOSINA	Non regulated
G04CA02	TAMSULOSINA	Non regulated
G04CA03	TERAZOSINA	Non regulated
G04CB01	FINASTERIDA	Non regulated
H01AB01	TIROTROPINA	Regulated
H01AC01	SOMATROPINA	Regulated
J01DH02	MEROPENEM	Regulated
J01DH03	ERTAPENEM	Non regulated
J01DH51	IMIPENEM E INHIBIDOR DE ENZIMA	Regulated
J07AL01	NEUMOCOCO ANTIGENOS POLISACARIDOS PURIFICADOS	Regulated
J07AL01//V08AA05	NEUMOCOCO ANTIGENOS POLISACARIDOS PURIFICADOS//EXTRANEAL	Regulated
J07AL02	VACUNA CONJUGADA NEUMOCOCICA	Non regulated
L01BA01	METROTEXATE	Regulated
L01XC02	RITUXIMAB	Regulated
L01XC03	TRASTUZUMAB	Regulated
L01XC07	BEVACIZUMAB	Regulated
L01XE01	IMATINIB	Regulated
L01XE02	GEFITINIB	Non regulated
L01XE05	SORAFENIB	Regulated
L01XE06	DASATINIB	Regulated
L01XE07	LAPATINIB	Regulated
L01XE08	NILOTINIB	Regulated
L01XE09	TEMSIROLIMUS	Regulated
L01XE11	PAZOPANIB	Regulated
L01XX04	SUNITINIB	Regulated
L01XX28	IMATINIB	Regulated
L01XX34	ERLOTINIB	Regulated
L03AB07	INTERFERON BETA-1A	Regulated
L03AB08	INTERFERON BETA-1B	Regulated
L04AA04	ANTITIMOCITO IMMUNOGLOBULINA (CONEJO)	Regulated
L04AA06	MICOFENOLICO ÁACIDO	Regulated
L04AA10	SIROLIMUS	Regulated
L04AA13	LEFLUNOMIDE	Regulated
L04AA18	EVEROLIMUS	Regulated
L04AA23	NATALIZUMAB	Regulated
L04AA24	ABATACEPT	Regulated
L04AA27	FINGOLIMOD	Regulated
L04AB01	ETANERCEPT	Regulated
L04AB02	INFLIXIMAB	Regulated
L04AB04	ADALIMUMAB	Regulated
L04AC07	TOCILIZUMAB	Regulated
M05BA04	ALENDRONICO ÁACIDO	Regulated
M05BA07	RISEDRONICO ÁACIDO	Non regulated
M05BA08	ZOLEDRONICO ACIDO	Regulated
N02BE01	PARACETAMOL	Regulated
N03AX09	LAMOTRIGINA	Regulated
N03AX12	GABAPENTIN	Regulated
N03AX14	LEVETIRACETAM	Regulated
N03AX16	PREGABALINA	Regulated
N05AH04	QUETIAPINA	Regulated
N05AN01	LITIO	Regulated
N05AX08	RISPERIDONA	Regulated
R03BA02	BUDESONIDA	Non regulated
R03DC03	MONTELUKAST	Regulated
S01EC03	DORZOLAMIDA	Non regulated
S01EC03//S01ED01	DORZOLAMIDA//TIMOLOL	Regulated
S01EC03//S01ED51	DORZOLAMIDA//TIMOLOL COMBINACIONES	Non regulated

$$ PI_{L} = \frac{{\mathop \sum \nolimits_{i = 1}^{I} P_{t,i} *Q_{t - 1,i} }}{{\mathop \sum \nolimits_{i = 1}^{I} P_{t - 1,i} *Q_{t - 1,i} }}, $$

where *P*_*t,i*_ is the price of active ingredient *i* in month *t* of the year and *P*_*t*−*1,i*_ is the price of active ingredient *i* in base year (September 2011). Prices and quantities were standardized to a common unit of concentration.

For each ATC, the common unit of concentration is the weight unit in which the concentration of the active ingredient is expressed. For example, Atorvastatin is sold in concentrations ranging from 10 to 40 mg, then, to make comparison possible among the different commercial presentations with this active ingredient, all prices and units sold by each ATC must be in a common unit. To achieve this, the concentration is multiplied by the total amount of minimal dispensed units (in this case, tablets) that are sold in each one of the commercial presentations, which gives the total amount of active ingredient (given in the common unit of concentration, in this case milligrams) per product.

The total units sold per commercial presentation are then multiplied by the respective total amount of active ingredient. The sum of these units is equal to the total amount of active ingredient sold in a specific time frame (month).

On the other hand, the market price reported in SISMED for each of the commercial presentations is divided by the total amount of active ingredient, to obtain the price per common unit of concentration per presentation.

In addition, *PI*_*L*_ was calculated by regulated and non-regulated ATC, and for the following therapeutical groups: immunosuppressant, insulins and oncology. Nominal sales were adjusted for inflation as measured by the Laspeyres price index to obtain the increase in real terms. The real value of expenditures, our proxy for quantities sold in the market, is thus obtained by removing the effect of price level changes from the nominal value of the time-series data.

All analyses were carried out in Colombian pesos. Figures were converted to US dollars using an exchange rate of 2976 COP/1 USD. It is important to note that the nominal exchange rate did not vary abruptly within the period 2011–2014 thus for pharmaceutical specialities that were imported prices do not reflect spurious exchange rate fluctuations. For 2015, although the exchange rate increased due to the oil price crisis, it did not affect our calculation because regulated prices were fixed in Colombian pesos and the formula used by the government included the average exchange rate of the year before. In sum, exchange rate fluctuations do not affect our price index calculation.

Laspeyres indexes hold previous period quantities fixed and may be biased because people may substitute away from drugs whose prices rise more rapidly. Accordingly, Laspeyres indexes tend to overstate inflation; however, such bias may be attenuated in Colombia because drug approvals are usually long and protracted. The administrative procedures can take up to 72 weeks. Also, the economic incentives work the other way around: new technologies entering the market are usually highly-priced.

## Main results

**Fig. 1:**
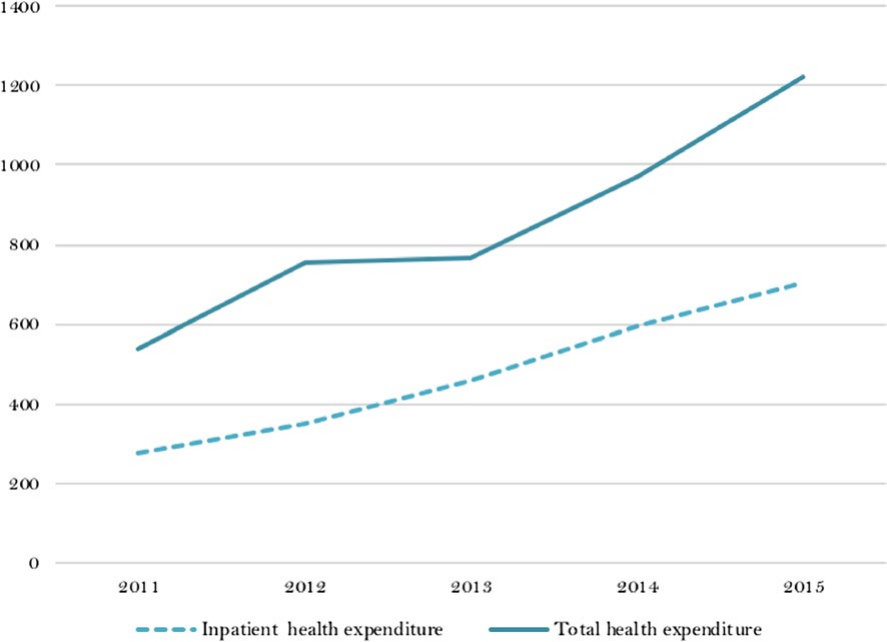
Health expenditure in No-POS drugs (US million) (Source: SISMED 2011–2015)

Despite direct price caps implemented in 2011, total No-POS drug expenditure grew significantly during 2012–2015. Total sales grew at a nominal annual rate of 23%, going from $538m in 2011 to $1224m in 2015 in US constant dollars (see Fig. 1). Total expenditure in the institutional market showed a similar pattern.

**Fig. 2:**
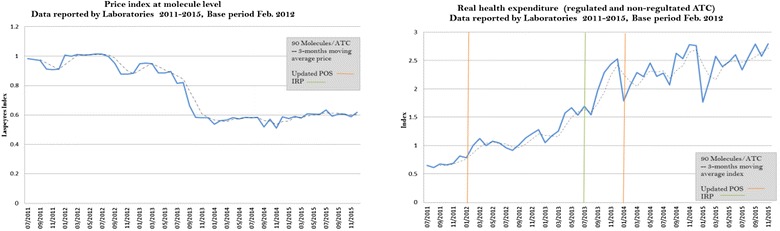
Price index and real health expenditure for regulated and non-regulated drugs (Source: Data are from July 2011 to December 2015, SISMED 2011–2015. Author’s estimations)

During the same period, prices decreased systematically until early 2014 (see Fig. 2). In the months after the first updating of the benefit package (POS), prices decreased about 43%. Later, after the IRP method was introduced in mid-2013, prices decreased at a faster rate until the second updating of the POS. In contrast, real pharmaceutical expenditure (quantities proxy) dramatically increased. In a 4-year period, quantities sold in the market doubled.

**Fig. 3:**
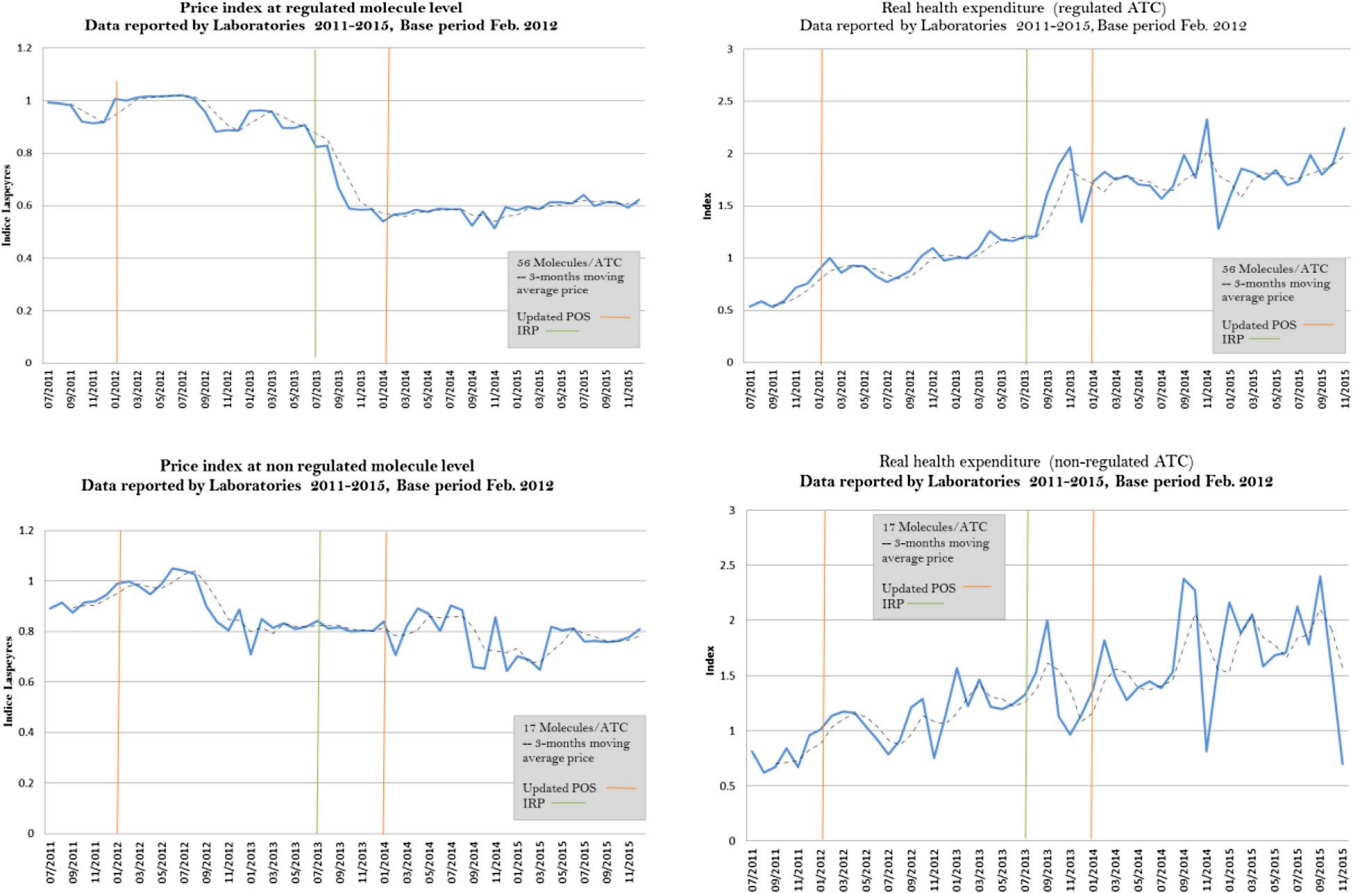
Price index and real health expenditure for regulated versus non-regulated drugs (Source: Data are from July 2011 to December 2015, SISMED 2011–2015)

The price index for regulated and non-regulated ATCs was also estimated. Figure 3 shows that the price index of regulated ATCs decreased 44% between 2012 and 2015, while that of non-regulated ATCs decreased 20% during the same period. Quantities sold increased in both groups. Quantities of regulated ATCs rose steadily. Non-regulated ATCs were more volatile.

**Fig. 4:**
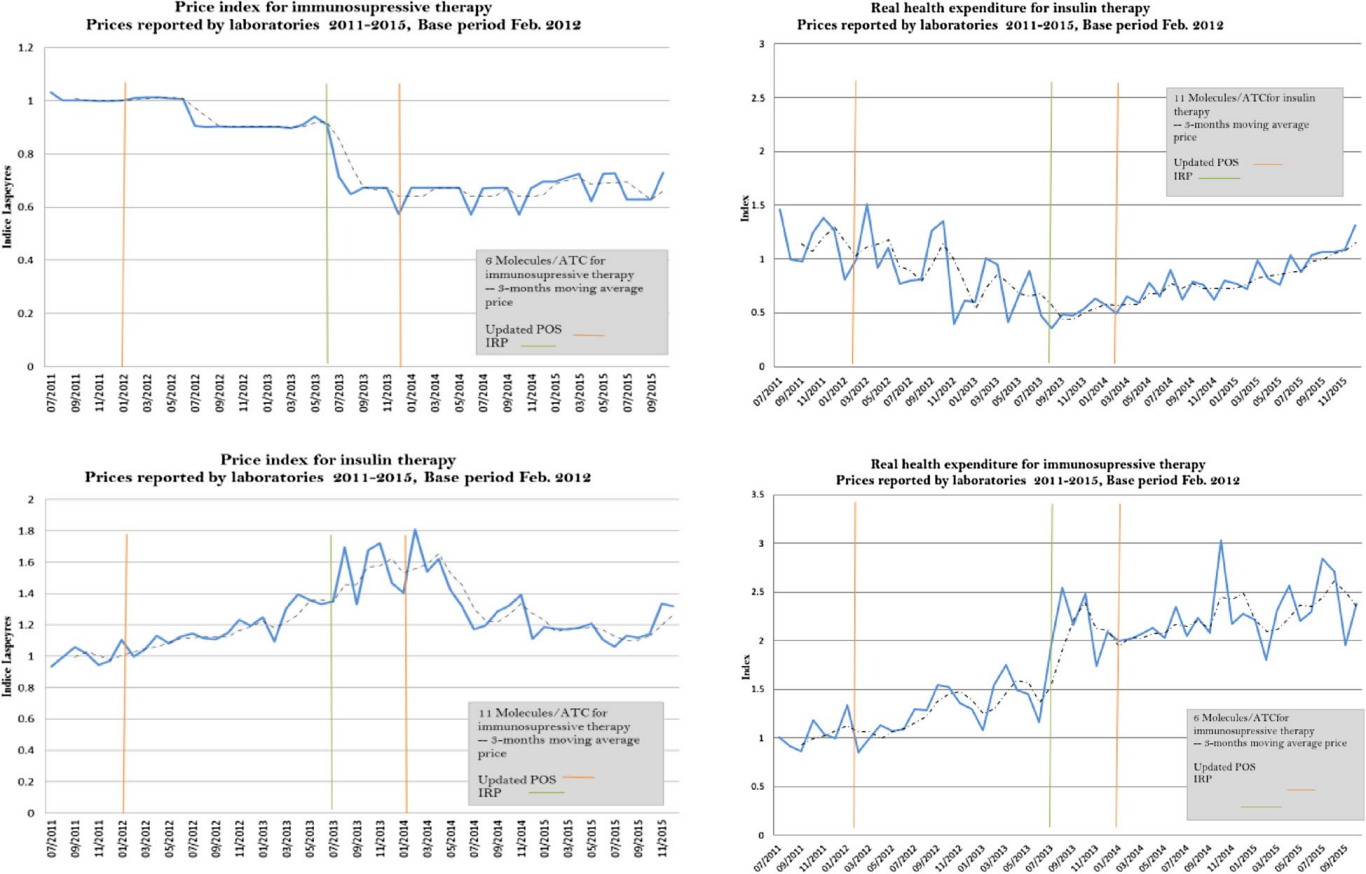
Price indexes and real health expenditure for immunosuppressive and insulin therapies. For immunosuppressive therapy indexes include the following ATC: L04AA23, L04AA24, L04AB01, L04AB02, L04AB04, and L04AC07. For insulin therapy indexes include the following ATC: A10AB01, A10AB04, A10AB06, A10AC01, A10AD01, A10AD01, A10AD04 and A10AE04 (Source: Data are from July 2011 to December 2015, SISMED 2011–2015)

The price index by therapeutic groups exhibit different patterns. For immunosuppressive ATCs, prices decreased 10% after the first POS update, and levelled off after the second. Units sold increased sharply during the whole period, 2011–2015 (see Fig. 4). All ATC included in this therapeutic group were subjected to a direct control price. In contrast, for insulins, prices increased around 60% until the second POS update. After this point, prices started to slightly decrease. Quantities sold did not increase in the period. This behaviour may be because this therapeutic class includes both regulated and non-regulated ATC.

## Discussion

Our descriptive analysis shows that, after price control measures were put in place, drug prices decrease about 43%, while real pharmaceutical health expenditure almost doubled. Similar results were obtained for therapeutic groups in which all ATC were regulated. However, if a therapeutic group includes regulated and non-regulated ATCs, results differed substantially. Overall, price control measures did not translate into lower No-POS expenditure. They likely induced an excess of demand.

The observed effect of ERP on prices is consistent with the extant literature on ERP in developed countries. In fact, most studies find a decrease in prices of regulated or new technologies [18, 25, 26]. However, the observed effect on pharmaceutical expenditure contrasts with some studies that report a decrease in pharmaceutical expenditure as a result of ERP [27].

The rapid increase in No-POS drugs expenditure motivated the Colombian government to adopt an aggressive price control strategy. The market response was a disproportionate increase in units sold. A part of such increase can be attributed to better access to drugs due to lower prices, and another part to an increase in marketing efforts by buyers. There is no data to tease apart these two purported effects.

Control policies reduce medicine prices. For the case of Colombia, Romero found that including a drug in the benefit package (POS) reduced its price by 14% and that introducing IPR reduced its price by 26% [19]. However, the effects upon total pharmaceutical expenditure are ambiguous. Among OECD countries, maximum price, IPR and health technologies reimbursement mechanism regulations were not found to be associated with cost containment of national health expenditures [28]. Evidence shows that pharmaceutical companies often diversify their portfolios into regulated markets and unregulated markets [21]. In addition, greater use of prescription drugs, replacement of older, cheaper drugs with new and more expensive ones, and the ever higher prices of retail prescription drugs also contribute to increase health expenditure [22].

Our study is limited in several ways. First the decomposition cannot be interpreted as a causal effect. Second, currently data is not audited (reporting is mandatory and a false report could bring serious fines). Third, even without the intention of reporting false data, typos and mistakes may still go undetected.

## Conclusions

The Colombian experience clearly shows that price controls do not necessarily decrease overall real pharmaceutical expenditures. Pharmaceutical expenditure is determined by variation in prices and quantities.^2^ A drug price regulation that does not consider a set of measures to strictly monitor (and eventually investigate and further control) quantities sold, is likely to fail in its objective of halting expenditure.

This is a crucial finding in the context of middle income countries like Colombia that still face challenges to guarantee financially sustainable universal healthcare coverage. While ERP may lower pharmaceutical prices, it may also spur an increase in the demand of regulated products, defying the cost control objective that motivated price regulation in the first place.

Increased demand of pharmaceutical products may be a positive thing, as long as the increase is the result of greater access to effective medicines rather than the result of induced demand by pharmaceutical producers. This is an important caveat in the context of middle-income countries where cost-effectiveness analyses and benefit package definition are relatively new, and not always legitimate, policy instruments.

## Footnotes

1 The CNPMDM is an inter-ministerial commission created for regulating pharmaceutical and medical device prices. The commission is formed by a representative of the President, the Minister of Trade and the Minister of Health and it relies on policy proposals developed by a technical advisory group based at the ministry of health.

2 Pharmaceutical expenditure is also determined by the structure of the market; that is, the share of generics for an active ingredient. Although we did not observe this variable in the paper it is worth acknowledging its importance.
